# The impact of tumor burden score on prognosis in patients after radical resection of hepatocellular carcinoma: a single-center retrospective study

**DOI:** 10.3389/fonc.2024.1359017

**Published:** 2024-11-01

**Authors:** Junzhang Huang, Ying Zhou, Suosu Wei, Yuntian Tang, Qiuhuan Zhang, Yi Tang, Wei Huang, Chongde Mo, Xiaofeng Dong, Jianrong Yang

**Affiliations:** ^1^ Department of Hepatobiliary, Pancreas and Spleen Surgery, Guangxi Academy of Medical Sciences, People’s Hospital of Guangxi Zhuang Autonomous Region, Nanning, China; ^2^ Department of Scientific Cooperation of Guangxi Academy of Medical Sciences, People’s Hospital of Guangxi Zhuang Autonomous Region, Nanning, China; ^3^ Department of Colorectal and Anal Surgery, People’s Hospital of Guangxi Zhuang Autonomous Region and Guangxi Academy of Medical Sciences, Nanning, China

**Keywords:** tumor burden, hepatocellular carcinoma, radical resection, survival, recurrence

## Abstract

**Purpose:**

This study examines the relationship between tumor burden score (TBS) and survival and recurrence following radical resection of hepatocellular carcinoma through a cohort study conducted in the Guangxi population of China.

**Methods:**

This cohort study eventually recruited 576 HCC patients undergoing radical resection of HCC in the People’s Hospital of Guangxi Zhuang Autonomous Region during 2013–2022. After determining the best threshold TBS, all cases were grouped to evaluate the relationship between TBS versus overall survival (OS) and cumulative recurrence. Using X-Tile software, the best threshold TBS to judge patient prognostic outcome following radical resection of HCC was 10.77.

**Results:**

Kaplan–Meier curve analysis revealed that patients with high TBS showed considerably decreased OS relative to the control group, accompanied by an increased recurrence rate. According to multivariate Cox proportional regression, the patients with high TBS were associated with poorer OS (HR = 2.56, 95% CI 1.64–3.99, *P* < 0.001) and recurrence-free survival (RFS) (HR = 1.55, 95% CI 1.02–2.35, *P* < 0.001).

**Conclusion:**

In patients undergoing radical resection for HCC, higher TBS was significantly related to shorter OS and RFS.

## 1 Introduction

Hepatocellular carcinoma (HCC) accounts for ~75% of primary liver cancer, with over 750000 cases annually. It ranks fifth among the most frequently seen malignant tumors and third among the factors leading to cancer-associated mortality worldwide (1–3). Surgery remains the cornerstone of treating patients with resectable HCC. Although only 20–30% of cases have resectable indications when diagnosed, surgical treatment is still the only potential choice for HCC treatment (4, 5). However, the early or late recurrence rate after surgery is high (6, 7), and according to some systematic reviews of large-scale data, the 5-year overall survival rate for patients with advanced HCC is still relatively low (8, 9).Therefore, research is needed to precisely estimate the long-time postoperative prognosis, thereby developing different classification systems and more appropriate risk stratification models. This will facilitate patient screening and improve long-time prognosis after HCC radical resection. In addition, this will help patients choose the most suitable treatment plan and conduct more targeted follow-up and post-surgery management to improve their survival rates and life quality.

In recent years, the American Joint Commission on Cancer (AJCC) and Barcelona Clinical Cancer Staging System are most commonly applied clinically (10). Previous research reports suggested multiple factors exerting adverse effects on the prognosis of patients with liver cancer, including morphological (i.e., maximum tumor size and number, collectively referred to as “tumor burden”) and pathological (such as microvascular infiltration, liver margin status, and liver capsule infiltration) features, and serum biomarkers (such as bilirubin, albumin, and alpha-fetoprotein (AFP)) (11, 12). Notably, tumor burden plays a critical role in predicting prognosis and stage determination (10). Recently, it has been considered a more accurate staging scheme than traditional Barcelona clinical liver cancer (BCLC) staging systems (13).

In 2018, the TBS was first proposed and proven useful in evaluating prognostic outcomes of cases receiving liver metastasis resection for colorectal cancer (14). In the past two years, Tsilimigras et al. proposed a method to calculate TBS using the Pythagoras theorem (α^2^+ β^2 =^ γ^2^, of which α = Maximum tumor diameter, β = tumor number, γ = TBS). This method continuously combines tumor size with quantity and is used for evaluating how TBS affects the prognostic outcome of HCC resection cases, demonstrating good prognostic differentiation and determining efficient prognosis (15). The ability to identify and determine the prognosis of patients with liver cancer facilitates selecting surgical candidates, providing appropriate postoperative monitoring and rehabilitation plans, and setting realistic goals for patients and nursing staff. However, only a few studies have been conducted on how TBS affects prognostic outcomes of cases undergoing HCC radical resection. Therefore, the current work focused on determining the impact of TBS on the prognostic outcome of patients with HCC undergoing radical resection.

## 2 Patients and methods

### 2.1 Objects of study and selection criteria

To build the retrospective cohort study, our group previously adopted an active health management platform, which was registered in the China Clinical Trial Registry (registration point http://www.chictr.org.cn/index.aspx; registration number: ChiCTR2200062446). It continuously includes patients with the pathological diagnosis of HCC who received radical resection in line with Chinese guidelines for diagnosing and treating HCC from May 2013 to March 2022 and confirmed the cases of liver cancer through the Health Management platform and big data platform of People’s Hospital of Guangxi Zhuang Autonomous Region, China (16).

**Figure 1:**
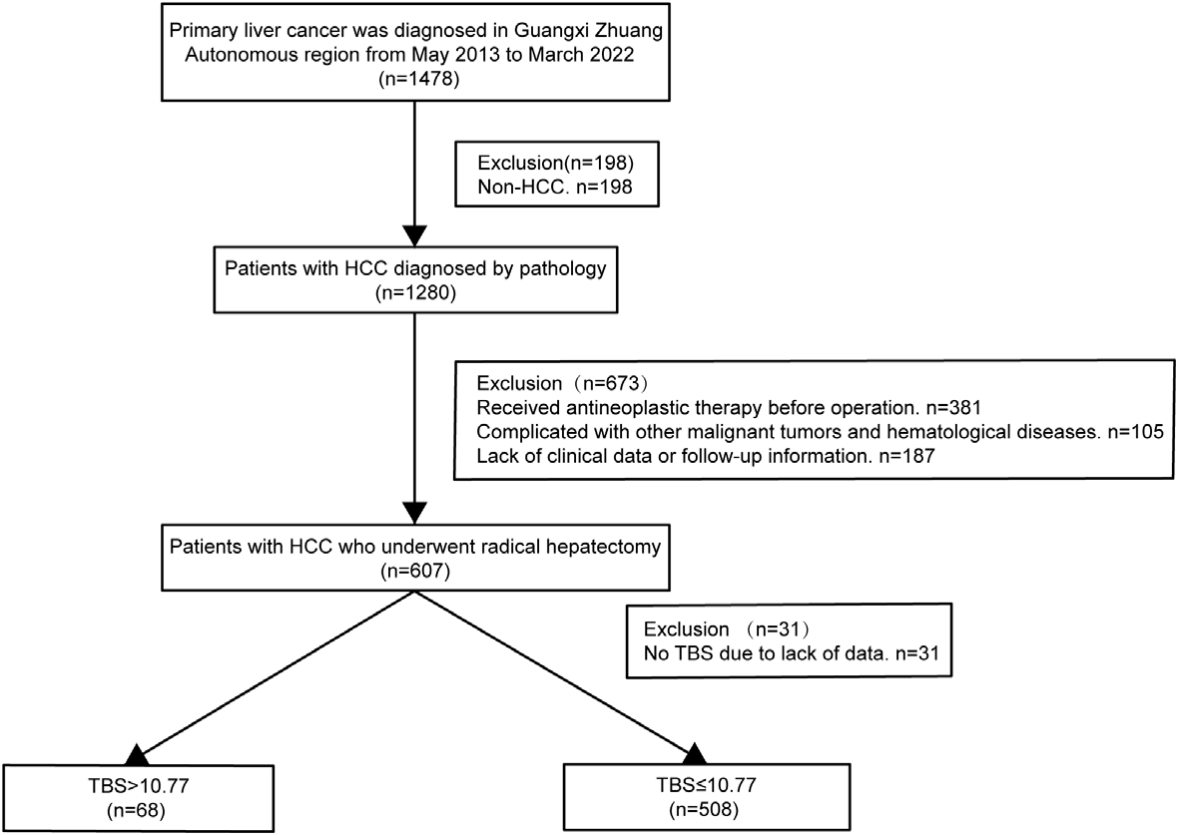
Flow chart for the selection of the study population. HCC, hepatocellular carcinoma; TBS, Tumor Burden Score.

Patients meeting indications for liver resection and undergoing radical liver resection, with histopathological confirmation of HCC, were included in the study. Following patients were eliminated: 1) Patients with insufficient clinical information. 2) Patients diagnosed with intrahepatic cholangiocarcinoma, hepatocellular cholangiocarcinoma, or additional system malignancies (like lung and colorectal cancers). 3) Patients developing severe organ dysfunction such as heart and lungs. 4) Patients with a history of preoperative transcatheter hepatic artery chemoembolization or radiofrequency ablation surgery. 5) Patients with a lack of complete clinical data (such as no information on pathological tumor size and quantity and no preoperative AFP measurement values). 6) Patients without sufficient follow-up data. **Figure 1** shows a flowchart of the procedure for patient screening. This research gained approval from the Ethics Committee of the Guangxi Academy of Medical Sciences and People’s Hospital of Guangxi and was performed following guidelines set out in the Declaration of Helsinki in 1975. Owing to the retrospective nature of the study, the data is anonymous, and patients did not provide written informed consent.

### 2.2 Definition

Overall survival (OS) is the period between the radical resection and death of the patient from any cause or last follow-up. Recurrence-free survival (RFS) is the duration between the radical resection and relapse or no relapse during the final follow-up period. Radical resection for HCC represents no macroscopic tumor thrombi in the large veins and bile ducts during surgery and no adjacent organ invasion. The distance between the tumor boundary and the liver cutting edge is ≥1cm, with no tumor lesions discovered during imaging examinations at 1–2 m postoperatively (17, 18). TBS is the distance between the Cartesian plane origin that comprises the maximum tumor size (x-axis) and tumor number (y-axis). TBS = (maximum tumor diameter)^2^ + (tumor number) (2, 15). The critical value for the patient’s serum AFP level threshold is 400 ng/mL; therefore, values <400 ng/mL represent low AFP levels, while values >400 ng/mL represent high AFP levels (19).

### 2.3 Interested variables and outcomes

Patient features include age, gender, history of liver cirrhosis, Body Mass Index (BMI), HBV-DNA, preoperative alpha-fetoprotein (AFP), Barcelona clinical liver cancer staging (BCLC), type of surgical approach (such as a minimally invasive procedure or open surgery), maximum tumor size and tumor number, TBS, microvascular infiltration, liver margin status (R0, R1), and liver capsule involvement.

OS was the primary endpoint of this study, while RFS was the secondary endpoint. The current study followed up through telephone, outpatient, and hospital electronic medical records between the final follow-up and June 30^th^, 2022. Relapse of HCC is the recurrence of new lesions completely meeting the diagnostic standards of HCC following radical resection. For patients with recurrence, post-surgery imaging findings (B-ultrasound, CT, MRI) are used to evaluate the HCC lesion recurrence (20, 21).

### 2.4 Statistical analysis

X-Tile software (17) (https://x-tile.Software.infoer.com/) was used to obtain the optimum threshold TBS for classifying all cases into two groups. Continuous variables were expressed as median and Quartile ranges (IQRs). Categorical and continuous data were compared using the Mann–Whitney U and Kruskal–Wallis tests, respectively. Classified data were expressed as total number and frequency, and differences between groups were analyzed using Chi-squared tests. Survival analysis was conducted using Kaplan–Meier analysis to analyze OS and RFS and compare them by log-rank test. The relation of TBS with postoperative prognosis of HCC cases was analyzed with a Cox proportional regression model, and hazard ratio (HR) and 95% confidence interval (CI) were determined. SPSS 27.0.1 software (IBM, Armonk, NY, USA) and R software (version 4.3.1, The R Project for Statistical Computing) was used for statistical analysis. *P* < 0.05 indicated statistical significance.

## 3 Results

### 3.1 Basic patient features

**Figure 2:**
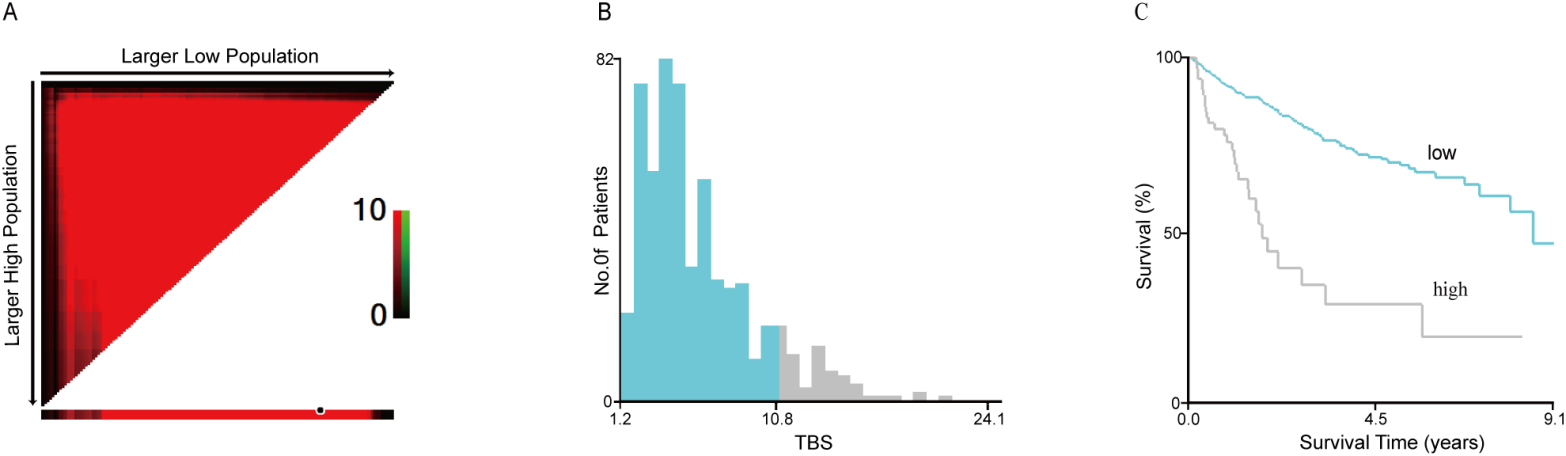
The cut-off value of TBS obtained by using the X-tile software was 10.77 where it was shown to have the strongest prognostic ability **(A–C)**. TBS, Tumor Burden Score.

Between May 2013 and March 2022, 1478 patients were diagnosed with primary liver cancer, and 576 were recruited in the current study. Based on X-Tile software analysis, the optimum TBS threshold to evaluate OS and RFS is 10.77 (**Figures 2A–C**). Based on this threshold, the study population was stratified (TBS ≤ 10.77: n = 508,88.2% and TBS > 10.77: n = 68,11.8%). For this cohort, the median age was 53.1 y old, with 475 patients (80.2%) being males. In terms of the patients’ nutritional status, those in the high TBS group are worse off than those in the low TBS group (low: 23.7 kg/m^2^ vs. high: 22.7 kg/m^2^; *P* = 0.014). Overall, 206 patients (35.8%) exhibited high AFP levels, and with the increase in TBS, the relative proportion of patients with high AFP levels significantly increased (low: 165, 32.5% vs. high: 41, 60.3%; *P* < 0.001). In addition, the relative open surgery rate (low: 341, 67.1% vs. high: 61, 89.7%; *P* < 0.001) and multiple tumors (low: 124, 24.4% vs. high: 27, 39.7%; *P* = 0.007) also increased with the increase of TBS. Compared to the low TBS group, the median maximum tumor nodule size in the high TBS group was significantly greater (low: 4.7 cm vs. high: 13.4 cm; *P* < 0.001). Patients with postoperative tumor pathology indicating liver capsule involvement have a higher relative proportion of patients with high TBS. (high: 24, 35.3% vs. low: 107, 21.1%; *P* = 0.009). In addition, in patients with microvascular infiltration of tumors, the relative difference between low and high TBS groups is more significant (low: 190, 37.4% vs. high: 50%, 73.5%; *P* < 0.001). According to the findings of this study, in the high TBS group, more patients are in Stage B based on the BCLC staging system (BCLC 0: n = 0,0% vs. BCLC B: n = 53, 77.9% vs. BCLC C: n = 15, 22.1%; *P* < 0.001). Nonetheless, TBS levels were not significantly related to age, gender, cirrhosis, HBV-DNA, and liver margin status (all *P* > 0.05) (**Table 1**).

**Table 1:** Demographics and patient characteristics.

Variables	Total (n=576)	TBS ≤ 10.77 (n=508)	TBS>10.77 (n=68)	P-value
Age, year	53.1 ± 11.7	53.4 ± 11.5	51.3 ± 13.1	0.171
BMI, kg/m²	23.6 ± 3.3	23.7 ± 3.3	22.7 ± 2.8	0.014
Sex, n (%)				0.085
Female	101 (17.5%)	84 (16.5%)	17 (25.0%)	
Male	475 (82.5%)	424 (83.5%)	51 (75.0%)	
Cirrhosis, n (%)				0.174
No	346 (60.1%)	300 (59.1%)	46 (67.6%)	
Yes	230 (39.9%)	208 (40.9%)	22 (32.4%)	
HBV-DNA, n (%)				0.472
No	345 (59.9%)	307 (60.4%)	38 (55.9%)	
Yes	231 (40.1%)	201 (39.6%)	30 (44.1%)	
AFP, ng/ml				<0.001
<400	370 (64.2%)	343 (67.5%)	27 (39.7%)	
≥400	206 (35.8%)	165 (32.5%)	41 (60.3%)	
Minimally invasive surgery, n (%)				<0.001
No	402 (69.8%)	341 (67.1%)	61 (89.7%)	
Yes	174 (30.2%)	167 (32.9%)	7 (10.3%)	
BCLC, stage				<0.001
0	49 (8.5%)	49 (9.6%)	0 (0.0%)	
A	463 (80.4%)	410 (80.7%)	53 (77.9%)	
B	64 (11.1%)	49 (9.6%)	15 (22.1%)	
Tumor size of largest nodule, cm	5.7 ± 3.7	4.7 ± 2.3	13.4 ± 2.7	<0.001
Tumor number, n (%)				0.007
Single	425 (73.8%)	384 (75.6%)	41 (60.3%)	
Multiple	151 (26.2%)	124 (24.4%)	27 (39.7%)	
Margin status, n (%)				0.072
R0	566 (98.3%)	501 (98.6%)	65 (95.6%)	
R1	10 (1.7%)	7 (1.4%)	3 (4.4%)	
Liver capsule involvement, n (%)				0.009
No	445 (77.3%)	401 (78.9%)	44 (64.7%)	
Yes	131 (22.7%)	107 (21.1%)	24 (35.3%)	
Microvascular invasion, n (%)				<0.001
No	336 (58.3%)	318 (62.6%)	18 (26.5%)	
Yes	240 (41.7%)	190 (37.4%)	50 (73.5%)	

Data are mean ± standard deviation, median (IQR) or N (%).

TBS, Tumor Burden Score; BMI, Body Mass Index; HBV, Hepatitis B Virus; AFP, Alpha-fetoprotein; BCLC, Barcelona Clinic Liver Cancer.

### 3.2 Survival analysis

**Figure 3:**
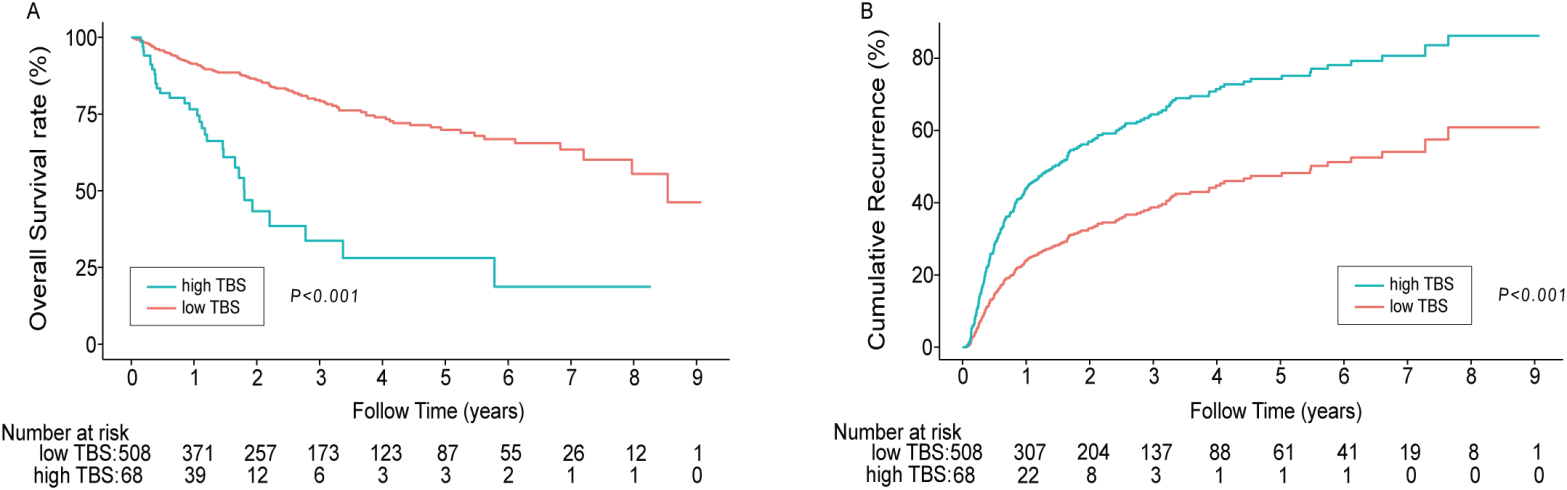
Kaplan–Meier curves demonstrating differences in OS **(A)** and recurrence **(B)** among patients with low TBS/high TBS. (*P*<0.001). TBS, Tumor Burden Score.

During the median 1.79 (IQR: 0.92–3.61) year follow-up period, 132 (22.92%) of the 576 patients died. The 1-, 3- and 5-year OS rates in the high TBS (>10.77) group were 76.58%, 28.08%, and 18.72%, respectively. These OS rates remarkably decreased compared with that of the low TBS (≤10.77) group (91.41%, 79.60%, and 68.92%) (P < 0.001). The median OS of the high TBS group (1.79 y) considerably decreased relative to the low TBS group (7.97 y) (P < 0.05). Similar results were obtained with regard to RFS. The 1-, 3-, and 5-year relapse rates in the high TBS group were 56.05%, 36.36%, and 36.36%, respectively, which significantly decreased relative to the low TBS group (76.50%, 61.21%, and 52.16%) (P < 0.001). Median RFS in the high TBS group (1.02 y) considerably decreased compared with that of the low TBS group (5.47 y) (**Figures 3A, B**).

### 3.3 Factors associated with RFS and OS

Roles of clinical pathological factors such as TBS in postoperative patient prognosis were analyzed using Cox regression. The univariate regression results suggested that TBS, BMI, tumor diameter, tumor number, preoperative AFP content, MVI, BCLC staging, and surgical approach were significantly related to OS (all *P* < 0.05). According to the multivariate regression, TBS (HR = 2.56, 95% CI 1.64–3.99, P < 0.001), BMI (HR = 0.94, 95% CI 0.88–0.99, *P* < 0.05), preoperative AFP levels (HR = 1.55, 95% CI 1.01–2.39, *P* < 0.05), and MVI (HR = 2.09, 95% CI 1.44–3.02, *P* < 0.001) demonstrated an independent relationship with OS. A high TBS level (>10.77) is significantly related to the shorter OS (**Table 2**). The univariable regression analysis revealed that TBS, age, HBV-DNA, tumor diameter, number of tumors, preoperative AFP level, liver margin status, MVI, and BCLC staging were significantly correlated with RFS (*P* < 0.05). Further, the multivariable regression suggested that TBS (HR = 1.55, 95% CI 1.02–2.35, *P* < 0.05), preoperative AFP levels (HR = 1.53, 95% CI 1.09–2.14, *P*<0.05), BCLC staging system (BCLC A: HR = 2.27, 95% CI 1.11–4.63, *P*<0.05; BCLC B: HR = 2.85, 95% CI 1.28–6.32, *P*<0.05) and MVI (HR = 1.79, 95% CI 1.34–2.39, *P*<0.001) were independent factors affecting the RFS prognosis. A high TBS level (>10.77) significantly correlates with a shorter RFS. Therefore, TBS score, preoperative AFP level, and the presence of MVI are common independent prognostic factors that significantly affect OS and RFS (**Table 3**).

**Table 2:** Univariate and multivariate analysis of the factors associated with OS.

Variables	Univariate analysis		Multivariate analysis	
	HR (95% CI)	P-value	HR (95% CI)	P-value
Age, year	0.99 (0.97, 1.00)	0.097	0.99 (0.97, 1.01)	0.329
BMI, kg/m²	0.93 (0.88, 0.98)	0.007	0.94 (0.88, 0.99)	0.022
Sex				
Female	1			
Male	1.45 (0.88, 2.39)	0.143		
Cirrhosis				
No	1			
Yes	1.01 (0.70, 1.46)	0.956		
HBV-DNA				
No	1			
Yes	1.18 (0.83, 1.66)	0.355		
AFP, ng/ml				
<400	1		1	
≥400	1.85 (1.31, 2.60)	<0.001	1.55 (1.01, 2.39)	0.047
Minimally invasive surgery				
No	1		1	
Yes	0.60 (0.37, 0.96)	0.034	0.67 (0.41, 1.10)	0.111
BCLC, stage				
0	1		1	
A	3.52 (1.12, 11.08)	0.032	2.63 (0.83, 8.34)	0.100
B	5.09 (1.50, 17.27)	0.009	3.22 (0.94,11.04)	0.063
Tumor size of largest nodule	1.14 (1.10, 1.19)	<0.001		
Tumor number				
Single	1			
Multiple	1.89 (1.32, 2.72)	<0.001		
Margin status				
R0	1			
R1	0.74 (0.10, 5.33)	0.769		
Liver capsule involvement				
No	1			
Yes	1.42 (0.97, 2.08)	0.069		
Microvascular invasion				
No	1		1	
Yes	2.60 (1.84, 3.69)	<0.001	2.09 (1.44, 3.02)	<0.001
TBS categories				
≤10.77	1		1	
>10.77	3.99 (2.64, 6.03)	<0.001	2.56 (1.64, 3.99)	<0.001

TBS, Tumor Burden Score; BMI, Body Mass Index; HBV, Hepatitis B Virus; AFP, Alpha-fetoprotein; BCLC, Barcelona Clinic Liver Cancer; OS, Overall Survival.

**Table 3:** Univariate and multivariate analysis of the factors associated with RFS.

Variables	Univariate analysis		Multivariate analysis	
	HR (95% CI)	P-value	HR (95% CI)	P-value
Age, year	0.98 (0.97, 1.00)	0.009	0.99 (0.98, 1.00)	0.056
BMI, kg/m²	1.02 (0.97, 1.06)	0.471	1.03 (0.98, 1.07)	0.223
Sex				
Female	1			
Male	1.24 (0.86, 1.80)	0.252		
Cirrhosis				
No	1			
Yes	1.05 (0.79, 1.40)	0.721		
HBV-DNA				
No	1			
Yes	1.48 (1.13, 1.94)	0.004		
AFP, ng/ml				
<400	1		1	
≥400	1.61 (1.22, 2.11)	<0.001	1.53 (1.09, 2.14)	0.013
Minimally invasive surgery				
No	1		1	
Yes	1.04 (0.76, 1.41)	0.826	1.05 (0.77, 1.44)	0.748
BCLC, stage				
0	1		1	
A	2.573 (1.27, 5.23)	0.009	2.27 (1.11, 4.63)	0.024
B	3.507 (1.60, 7.70)	0.002	2.85 (1.28, 6.32)	0.010
Tumor size of largest nodule	1.09 (1.06, 1.13)	<0.001		
Tumor number				
Single	1			
Multiple	1.94 (1.46, 2.59)	<0.001		
Margin status				
R0	1			
R1	2.59 (1.06, 6.31)	0.037		
Liver capsule involvement				
No	1			
Yes	1.35 (1.00, 1.84)	0.053		
Microvascular invasion				
No	1		1	
Yes	2.13 (1.62, 2.81)	<0.001	1.79 (1.34, 2.39)	<0.001
TBS categories				
≤10.77	1		1	
>10.77	2.11 (1.42, 3.14)	<0.001	1.55 (1.02, 2.35)	0.039

TBS, Tumor Burden Score; BMI, Body Mass Index; HBV, Hepatitis B Virus; AFP, Alpha-fetoprotein; BCLC, Barcelona Clinic Liver Cancer; RFS, Recurrence-free Survival.

### 3.4 Subgroup analysis

**Figure 4:**
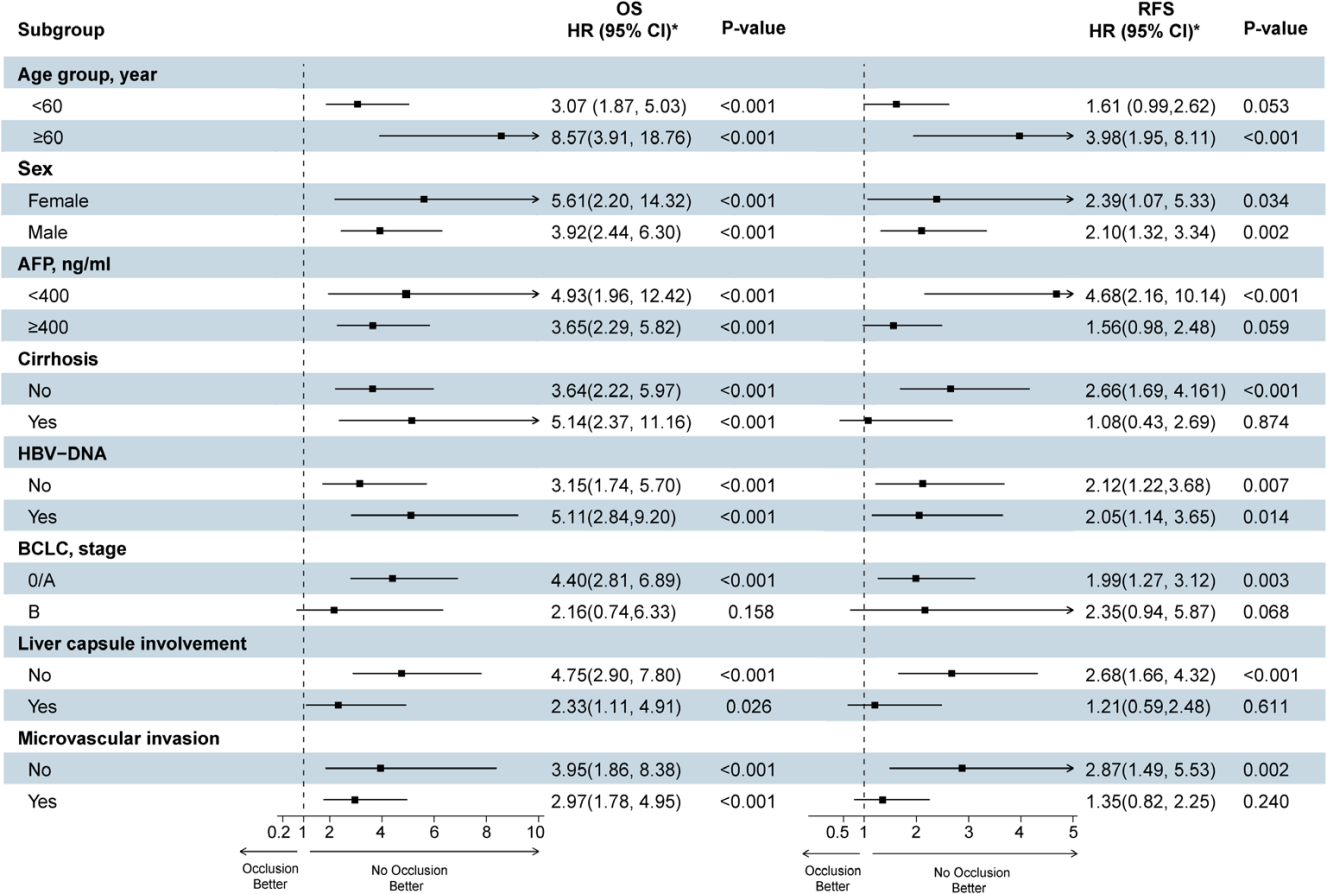
Forest plots show the effect of high TBS on survival and recurrence after surgery for hepatocellular carcinoma (The TBS ≤ 10.77 group was reference group). TBS, Tumor Burden Score; HBV, hepatitis B virus; AFP, alpha-fetoprotein; BCLC, Barcelona Clinic Liver Cancer; RFS, recurrence-free survival; OS, overall survival. Each stratification was adjusted for all the factors (TBS, Age, Sex, AFP, Cirrhosis, HBV-DNA, BCLC, Liver capsule involvement, Microvascular invasion) except the stratification factor itself.

To further study the role of TBS score in predicting HCC prognostic outcome, we performed a subgroup analysis of age, sex, AFP level, MVI, HBV-DNA, liver cirrhosis, and BCLC staging, Liver capsule involvement, and depicted a forest plot (**Figure 4**). Notably, even after adjusting for these variables, TBS was closely associated with overall relapse risk and death in HCC cases with radical resection.

### 3.5 Sensitivity analysis using PSM

To minimize the possible confounding bias, we performed PSM in high AFP and low TBS, the characteristics of the two group after PSM was showed in **Supplementary Table S1**. Kaplan-Meier analysis performed in the after PSM data showed that the relationship between TBS and the OS and RFS in patients with hepatocellular carcinoma after radical resection was consistent to that prior to PSM, which validated our results (**Supplementary Figure S1**).

## 4 Discussion

Currently, HCC displays poor treatment prognosis, and converse to the decrease in the mortality rate of other common cancers, the mortality rate of HCC increases by ~2–3% annually (22). Epidemiological data predict that it will continue to rise at least until 2030 (23). In China, the liver cancer mortality rate is higher in rural areas than in urban regions, and the western areas exhibit higher mortality rates compared to the eastern and central regions. This elevated mortality in rural and western areas is partly associated with the higher incidence of liver cancer (24). Among various treatment options, surgery remains the first choice for treating HCC among numerous treatment options. However, in several cases, owing to the lack of transplanted organs, hepatectomy takes precedence over liver transplantation (25). However, the recurrence and mortality rates of patients undergoing hepatectomy are still high (26). Traditionally, the survival of patients with HCC is usually assessed from the diagnosis or treatment of the disease, which often underestimates the prognostic outcome of the patients who have undergone surgery (27, 28). TBS integrates tumor size with quantity for evaluating overall tumor burden (15, 29). Because the method of calculating tumor burden is continuous rather than dichotomous, the prediction of results is more accurate than that possible using the current Milan standard (14), incorporating TBS allows for better risk stratification and identification of patients who require closer monitoring, thereby providing improved prognostic value (30). However, TBS was initially developed to evaluate the prognostic outcome of colorectal cancer following liver metastasis resection, but recently it has been extensively applied in evaluating the prognostic outcome of resectable HCC cases and demonstrates notable advantages (13, 14). This study aims to confirm whether the TBS score can become crucial in influencing the prognostic outcome of cases after radical resection of HCC to promote its clinical application. The observations of the current study revealed that TBS and other clinical pathological indicators have significant independent predictive value for the prognostic outcome of cases involving HCC radical surgery. For patients undergoing radical resection of HCC, high TBS is significantly related to shorter OS and RFS.

This retrospective cohort study used the X-Tile bioinformatics tool to determine the best threshold TBS of 10.77 so that all cases were classified into high (>10.77) or low TBS groups (≤10.77) (31). This is different from the cutoff value previously reported by Tsilimigras et al. (15), considering the differences arising from different patient characteristics. From the distribution of clinical characteristics of the patients, we observed that the operation being laparotomy, high preoperative AFP levels, BCLC stage of stage B, greater tumor diameter, multiple tumors with hepatic capsule invasion or the MVI, and higher TBS. Studies showed these factors are related to poor postoperative patient outcomes (32). In particular, patients with elevated AFP (33), MVI (34), or hepatic capsule invasion (35) have a higher risk of recurrence, and these factors are closely related to treatment outcome and poor prognosis. Notably, only some patients with high TBS have liver cirrhosis (32.4%), while patients with low TBS have a higher proportion of liver cirrhosis (40.9%). The possible reason for this is that patients with higher tumor burden must demonstrate better potential liver function before they are identified as candidates for surgical resection to minimize the risk of postoperative liver failure (36). Based on univariate and multivariate analyses, high TBS levels showed a higher risk of shortening OS and RFS, which independently predicted the prognosis of the patients with HCC receiving radical hepatectomy. Based on univariable/multivariable regression, higher TBS levels were positively correlated with shortened OS and RFS. High levels of TBS independently predicted the prognosis of the patients with HCC who underwent radical hepatectomy. In addition, preoperative high levels of AFP and MVI showed an independent relationship to OS and RFS. The Kaplan–Meier curve shows that compared with the high TBS group, the low TBS group demonstrated significantly extended OS and RFS. This finding agreed with the conclusion by Tsilimigras et al. The team used TBS combined with AFP levels to evaluate prognostic outcomes in cases undergoing radical liver cancer resection. The results showed that at low AFP levels, the high TBS group showed significantly decreased 5-year OS rates relative to the low and middle TBS groups (47.7% vs. 68.0% OS, P < 0.001) (15). Similarly, at higher AFP levels, the decreased TBS is related to superior OS (15). Another recently performed large retrospective study found that the peak risk of intrahepatic relapse in the high TBS score group approximately doubled than that of the low TBS group, which showed an independent relationship to a high risk of recurrence (27). This suggests that patients will experience a significantly increased early and extrahepatic relapse risk during surgery for HCC with high TBS scores. Therefore, higher relapse risk and more invasive relapse modes are recognized after the operation, and corresponding measures should be taken. In recent years, several articles confirmed the effective application of TBS in the prognosis prediction of cases receiving radical resection of HCC (37, 38). Such research regarding the prognostic significance of TBS in patients with HCC consistently shows that compared with low TBS, high TBS is closely related to poor clinicopathological features and adverse complications, suggesting poor prognosis after treatment.

Indeed, tumor morphology and the tumor microenvironment are critical factors that determine the biology and invasiveness of tumors. A high TBS indicates a larger tumor diameter, and as the tumor expands, the incidence of microvascular invasion continues to rise, particularly in tumors exceeding 10 centimeters (39). Furthermore, a growing body of evidence indicates that the tumor microenvironment—including cancer-associated fibroblasts, tumor-associated macrophages, oncogenic signaling pathways, tumor-associated extracellular matrix, activated tyrosine kinases, regulatory immune cells that support tumor progression, tumor-associated exosomes, inflammation-related cells, ligand-receptor interactions, immune escape mechanisms, and endothelial cell-mediated angiogenesis—plays a critical role in tumor progression. This creates a vicious cycle between cancer cells and the tumor microenvironment (40). The tumor microenvironment presents a promising target for therapy, as it plays a crucial role in regulating tumor-specific immune responses and enhancing the survival rates of liver cancer patients, particularly those with HCC (40).

However, the current study is somewhat different from others because its focus was to elucidate the role of TBS in the prognostic outcome of cases receiving radical resection of HCC. The subgroup analysis included age, sex, AFP level, MVI, mode of operation, HBV-DNA, liver cirrhosis, hepatic capsule involvement, and BCLC staging. Considering the group with TBS ≤ 10.77 as the control group, the significance of TBS in prognosis was confirmed and more intuitively explained. The results showed that TBS > 10.77 was the risk factor related to OS and RFS for all age groups, sex, BCLC stage, preoperative AFP level, no liver cirrhosis, HBV-DNA, mode of operation, MVI, and hepatic capsule involvement. TBS score demonstrates the advantages of simple acquisition, economy, and good repeatability, resulting in stability and intuitive nature of the results. This offers objective and valuable prognostic data for HCC cases receiving radical resection. Therefore, using the TBS score assists clinicians in the early detection of potentially poor prognostic outcomes in HCC cases and strengthens the dynamic monitoring of patients to adopt individualized adjuvant therapeutic measures. Furthermore, the etiology of liver cancer in China is closely linked to hepatitis B virus infection. This study indicates that HBV-DNA is a risk factor for OS and RFS. Implementing vaccination and antiviral treatments to reduce risk factors such as HBV and HCV can help decrease the TBS at diagnosis (41).

This study demonstrates the following merits: First, this study further analyzed the correlation between prognosis and TBS in liver cancer patients from the Guangxi region of China. Compared to previous studies in the same area, this research has a larger scale and longer follow-up time, making the conclusions more convincing. Additionally, it provides data support for the diagnosis and treatment prognosis of liver cancer in different countries and regions. Secondly, the cut-off value was optimized using X-Tile software. Thirdly, we excluded patients who had received antineoplastic therapies other than surgery to maintain the consistency of baseline data and minimize interference from other treatment methods. Finally, subgroup analysis confirmed that TBS possesses significant predictive value. Nonetheless, this study also has some limitations. First, although the study selected patients strictly according to the established criteria, an inevitable selection bias was present. Second, this study obtained data from one center, focusing on cases undergoing surgery. Third, the cutoff value of TBS needs to be verified from more prospective articles. Finally, the cases in this research are from China, and the major cause of the disease is associated with hepatitis B virus infection. Consequently, the current findings lack scientific guidance in HCC cases primarily resulting from alcoholic liver disease. Therefore, relevant research is needed, including data from multiple countries and populations worldwide. Despite these limitations, this study provides interesting data regarding how TBS affects HCC prognosis.

In summary, TBS is an important index for predicting prognostic outcomes in HCC cases. For cases undergoing radical resection of HCC, higher TBS is significantly associated with shorter OS and RFS. Tumor morphology (tumor burden) affects evaluating the prognostic outcome of cases undergoing hepatectomy.

## Data Availability

The raw data supporting the conclusions of this article will be made available by the authors, without undue reservation.

## Supplementary material

Supplementary Figure S1 Kaplan-Meier analysis performed in the after PSM data. **(A)** Survival rate of group (low TBS vs. high TBS, *P*<0.05); **(B)** Recurrence-free survival of group (low TBS vs. high TBS, *P*=0.74). TBS, Tumor Burden Score.
