# Supplementary figures and images for: The impact of tumor burden score on prognosis in patients after radical resection of hepatocellular carcinoma: a single-center retrospective study
Source: Front Oncol. 2024 Nov 1;14:1359017. doi: 10.3389/fonc.2024.1359017 (PMC11563962; doi:10.3389/fonc.2024.1359017)

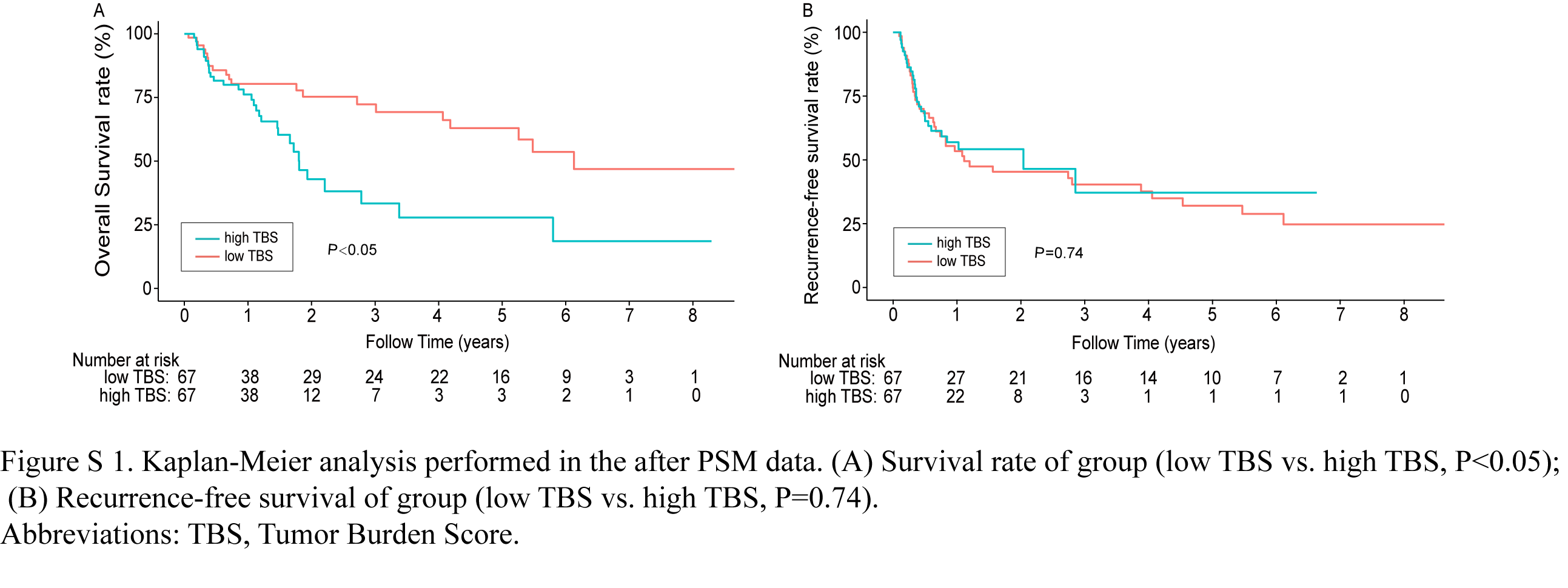

Supplement: Supplementary Figure S1 — Kaplan-Meier analysis performed in the after PSM data. (A) Survival rate of group (low TBS vs. high TBS, P<0.05); (B) Recurrence-free survival of group (low TBS vs. high TBS, P=0.74). TBS, Tumor Burden Score. [file Image1.tif]
