# Supplementary material for: The impact of tumor burden score on prognosis in patients after radical resection of hepatocellular carcinoma: a single-center retrospective study
Source: Front Oncol. 2024 Nov 1;14:1359017. doi: 10.3389/fonc.2024.1359017 (PMC11563962; doi:10.3389/fonc.2024.1359017)
Supplement: Supplementary file 2 [file Table1.docx]

| Supplementary Table 1 Demographics and patient characteristics with after PSM | | | | |
| --- | --- | --- | --- | --- |
| Variables | Total(n=134) | TBS≤10.77(n=67) | TBS>10.77(n=67) | P-value |
| Age, year | 50.6 ± 12.4 | 49.8 ± 11.8 | 51.5 ± 13.1 | 0.421 |
| BMI, kg/m² | 23.0 ± 3.6 | 23.3 ± 4.1 | 22.6 ± 2.9 | 0.271 |
| Sex, n (%) |  |  |  | 0.699 |
| Female | 37 (27.6%) | 20 (29.9%) | 17 (25.4%) |  |
| Male | 97 (72.38%) | 47 (70.1%) | 50 (74.6%) |  |
| Cirrhosis, n (%) |  |  |  | 0.852 |
| No | 92 (68.7%) | 47 (70.1%) | 45(67.2%) |  |
| Yes | 42 (31.3%) | 20 (29.9%) | 22 (32.8%) |  |
| HBV-DNA, n (%) |  |  |  | 1.000 |
| No | 76 (56.7%) | 38 (56.7%) | 38 (56.7%) |  |
| Yes | 58 (43.3%) | 29 (43.3%) | 29 (43.3%) |  |
| AFP, ng/ml |  |  |  | 0.001 |
| <400 | 14 (10.4%) | 1 (1.5%) | 13 (19.4%) |  |
| ≥400 | 120 (89.6%) | 66 (98.5%) | 54 (80.6%) |  |
| Minimally invasive surgery, n (%) |  |  |  | 1.000 |
| No | 120 (89.6%) | 60 (89.6%) | 60 (89.6%) |  |
| Yes | 14 (10.4%) | 7 (10.4%) | 7 (10.4%) |  |
| BCLC, stage |  |  |  | 0.463 |
| 0 | 1 (0.7%) | 1 (1.5%) | 0 (0.0%) |  |
| A | 105 (78.4%) | 53 (50.5%) | 52 (77.6%) |  |
| B | 28 (20.9%) | 13 (19.4%) | 15 (22.4%) |  |
| Tumor size of largest nodule, cm | 9.8 ± 4.3 | 6.2 ± 2.4 | 13.3 ± 2.5 | <0.001 |
| Tumor number, n (%) |  |  |  | 0.859 |
| Single | 82 (61.2%) | 42 (62.7%) | 40 (59.7%) |  |
| Multiple | 52 (38.8%) | 25 (37.3%) | 27 (40.3%) |  |
| Margin status, n (%) |  |  |  | 1.000 |
| R0 | 131 (97.8%) | 66 (98.5%) | 65 (97.0%) |  |
| R1 | 3 (2.2%) | 1 (1.5%) | 2 (3.0%) |  |
| Liver capsule involvement, n (%) |  |  |  | 0.592 |
| No | 84 (62.7%) | 40 (59.7%) | 44 (65.7%) |  |
| Yes | 50 (37.3%) | 27 (40.3%) | 23 (34.3%) |  |
| Microvascular invasion, n (%) |  |  |  |  |
| No | 38 (28.4%) | 20 (29.9%) | 18 (26.5%) | 0.848 |
| Yes | 96 (71.6%) | 47 (70.1%) | 49 (73.1%) |  |

Data are mean ± standard deviation, median (IQR) or N (%).

Abbreviations: TBS, Tumor Burden Score; BMI, Body Mass Index; HBV, Hepatitis B Virus; AFP, Alpha-fetoprotein; BCLC, Barcelona Clinic Liver Cancer.
